# Use of serology and real time PCR to control an outbreak of bovine brucellosis at a dairy cattle farm in the Nile Delta region, Egypt

**DOI:** 10.1186/s13620-016-0062-9

**Published:** 2016-02-24

**Authors:** Mayada Gwida, Maged El-Ashker, Falk Melzer, Mohamed El-Diasty, Mohamed El-Beskawy, Heinrich Neubauer

**Affiliations:** 1 Department of Hygiene and Zoonoses, Faculty of Veterinary Medicine, Mansoura University, Mansoura, 35516 Egypt; 2 Department of Internal Medicine and Infectious Diseases, Faculty of Veterinary Medicine, Mansoura University, Mansoura, 35516 Egypt; 3 Friedrich-Loeffler-Institute (FLI), Institute of Bacterial Infections and Zoonoses, 07743 Jena, Germany; 4 Animal Health Research Institute-Mansoura Provincial Laboratory, Mansoura, Egypt

**Keywords:** Brucellosis, Cattle, Serology, RT-PCR, Public Health, Egypt

## Abstract

**Background:**

Bovine brucellosis remains one of the most prevalent zoonotic infections affecting dairy cattle in developing countries where the applied control programs often fail. We analyzed the epidemiologic pattern of bovine brucellosis in a dairy cattle herd that showed several cases of abortions after regular vaccination with RB51 (*B. abortus* vaccine). In 2013 thirty dairy cows, from a Holstein-Friesian dairy herd with a population of 600 cattle, aborted five months post vaccination by a regular RB51 vaccine. Blood samples were drawn from milking cows and growing heifers, as well as heifers and cows pregnant up to 6 months. These samples were collected in June 2013 (*n* = 257) and May 2014 (*n* = 263) and were tested by real time (rt)-PCR as well as serological tests, in particular Rose Bengal Test (RBT), Enzyme-Linked Immunosorbent Assays (ELISA) and Fluorescence Polarization Assay. Tissue specimens were also collected from an aborted fetus and cultured. Isolates were subjected to bacteriological typing tests at the genus and species levels.

**Results:**

Five months post vaccination with RB51 vaccine, *Brucella (B.)* DNA was detected in blood samples of cows by rt-PCR. The serological tests also revealed the spread of *Brucella* field strains within the herd in 2013. Four *Brucella* isolates were recovered from specimens collected from the aborted fetus. These isolates were typed as follows: one *B. abortus* RB51 vaccine strain and three isolates of *B. abortus* field strain. The seropositive cows with positive rt-PCR might indicate an infection by the *Brucella* field strain; while the positive rt-PCR results from seronegative animals may either be due to circulating RB51 vaccine DNA in vaccinated animals or to circulating field strain in infected animals before seroconversion.

**Conclusion:**

The results herein suggest that PCR can be a good supplementary tool in an outbreak situation, if an assay is available that can differentiate vaccine and field strains with a high analytical sensitivity. We recommend using RBT and ELISA in parallel in outbreak situations, to identify as many infected animals as possible during the initial screenings. This test procedure should be repeated for at least three successive negative tests, with one month interval.

## Background

Brucellosis, caused by *Brucella* species, is considered one of the most common bacterial zoonotic infections worldwide. The infection is endemic in many geographic areas including the Mediterranean and Middle East regions, Indian subcontinent, Mexico and parts of central and South America [1, 2]. The disease is a major cause of direct economic loss and impedes trade among countries [3]. The estimated annual economic loss due to brucellosis was about 60 million Egyptian pounds in 1995 [4]. The diagnosis of *Brucella* infection is usually based on clinical presentation, microbiological culture and demonstration of specific antibodies [5]. Despite being the more commonly used technique for the initial screening of the infection, serological tests have limitations because not all infected animals produce detectable levels of antibodies, and false-positive results are seen due to cross-reactivity with antigens of other bacterial species [6]. The sensitivity of bacteriological culture usually depends on the viability and number of *Brucella* in the sample, as well as contamination of the sample with other bacteria [7]. Conversely, culture methods are time-consuming and the handling of microorganisms is hazardous [5]. In order to overcome most of these difficulties, PCR and real-time (rt)-PCR assays have been employed for diagnosis and molecular typing of *Brucella* species. Various PCR assays targeting different gene loci have been successfully used for diagnosis of brucellosis [8–12]. Species identification and sub-typing of *Brucella* isolates are very important not only for epidemiologic surveillance, but also for investigation of outbreaks in brucellosis endemic regions [13, 14]. Multiple-Locus Variable number tandem repeat Analysis (MLVA) is a useful tool for identifying and genotyping of *Brucella* isolates and data could be used for epidemiological trace back investigations [15, 16].

Bovine brucellosis remains one of the most common zoonoses in Egypt, and causes great constraints to the Government in improving animal productivity. Although nearly a century has passed since the first description of *Brucella* in Egypt, it has not been possible to eradicate this infection. The enforcement of control measures for brucellosis in Egypt (test and slaughter, S19 vaccination) have led to a reduction of *B. abortus* incidence in cattle [17]. However, most of these endeavors are still not conclusive [17, 18]. Despite being endemic, little is known about the epidemiological situation of brucellosis among Egyptian dairy herds. Indeed, *Brucella* infection remains under-diagnosed and often underestimated. In general, the control programs of *Brucella* infection in animals relies mainly on vaccination with live attenuated *B. abortus* strain 19. This provides good levels of protection against *B. abortus* in cattle and *B.melitensis* Rev. 1is used in sheep and goats [19, 20]. When administered correctly, the two vaccines can protect livestock from brucellosis, but the vaccines still have a drawback as they retain pathogenicity and sometimes cause abortion in vaccinated animals [21] and debilitating illness in humans [22–24]. In 2006, RB51, a mutant vaccine rough strain that is devoid of the lipopolysaccharide O-side chain was developed [25]. Cattle vaccinated with this kind of vaccine remained negative in conventional brucellosis serological tests [26]. This vaccine was suggested to be more appropriate than *B. abortus* S19 for the control and eradication programs that relied on serological testing and removal of positive animals [27].

For the effective monitoring of bovine brucellosis, it is imperative to have reliable tests to differentiate between vaccine and field strains. Thus, many molecular approaches have been developed to detect vaccine strains [28, 29]. Although extensive reports of animal brucellosis in Egypt are currently available, the epidemiologic situation of this infection is still ambiguous and needs further investigations. The purpose of this study was to analyze the epidemiologic pattern of bovine brucellosis in a well managed dairy farm in Egypt through a two year study period.

## Methods

### Farm description and problem identification

In 2013, a string of abortions (*n* = 30) was reported in a commercial dairy farm with a stock population of 600 Holstein-Friesian cattle. The farm’s records indicated that the farm was free from brucellosis since 2008. All animals tested negative using Rose Bengal Test (RBT) and Buffered Acidified Plate Agglutination Test (BAPAT) and the farm was considered an “ideal” dairy farm. This means all effective health care programs were enforced. All “good dairy farming” practices for animal health are practiced under the following guidelines: preventing the entry of disease onto the farm, having an effective herd health management program in place, and using all chemicals and veterinary medicines as directed. The farm is located in Gamasa City on the coast of the Mediterranean Sea, Dakahlia Governorate, Egypt.

The main target groups of this study were milking cows, growing heifers plus heifers and cows being pregnant up to 6 months. The age of these animals ranged between 6 - to -18 months for heifers, and 2- to-5 years for pregnant and non pregnant cows. A routine investigation for *Brucella* infection was performed with cattle at this farm in December 2012 using RBT and BAPAT and all cattle tested negative. For this reason, all animals (except those being pregnant > 3 months; *n* = 80) were vaccinated with RB51 (*B. abortus* vaccine, Professional Biological Company, USA) at the beginning of January 2013 according to the instructions of the manufacturer. Thirty cows aborted in June 2013. As a result of this, the pregnant heifers (<6 months) and all cows (except those pregnant in the third trimester) were sampled for detailed serological and molecular investigations of *Brucella* infection. With the exception of abortion, no other clinical signs appeared in these animals during sampling. All animals on the farm remained clinically healthy without any detectable illness. During the period of abortions, the farm veterinarian exhibited some brucellosis related signs of infection including undulated fever, back and joint pain. 

### Samples collection

#### Blood samples

The target groups of animals were sampled twice: in June 2013 (*n* = 257) and May 2014 (*n* = 263). Each time, ten ml of blood was collected through jugular vein puncture into vacutainer tubes without anticoagulant for separation of blood serum. The collected sera were serologically examined by RBT, Enzyme-Linked Immunosorbent Assays (ELISA) and Fluorescence Polarization Assay (FPA) at Friedrich-Loeffler-Institute (FLI), OIE reference laboratory in Jena. RBT was performed as previously described in the Manual of Standards for Diagnostic Tests and Vaccines [6] using antigen obtained from Institute Pourquier, France. Positive and negative control sera were the German national reference sera standardized according to OIE. The ELISA was performed and results were interpreted according to the instructions of the manufactures using IDEXX™ *Brucella* ELISA kit (Montpellier SAS, France). FPA was done and results were interpreted according to the instructions of the manufacturer (Diachemix, Whitefish Bay, WT, USA). A blood sample was also drawn from the farm veterinarian and was tested by RBT, ELISA, FPA and rt-PCR. Cultural procedures and molecular diagnostic assays were also performed at FLI, Jena, Germany. An informed consent for *Brucella* investigation was given by the owners. All procedures were performed in accordance with the principles and specific guidelines presented in the Guidelines for the Care and Use of Agricultural Animals in Research and Teaching, 3^rd^ ed. (http://www.fass.org/docs/agguide3rd/Ag_Guide_3rd_ed.pdf), and those of Mansoura University Animal Care and approved by its Ethical Committee.

#### Tissue specimens

Abomasal contents, liver, kidney and spleen were collected from an aborted fetus in June 2013 for isolation of *Brucella* organism. All tissue samples were cooled immediately after being collected, and were immediately transported to the laboratory. The collected specimens were cultured and subjected to the commonly used bacteriological typing tests at the genus and species (biovar) levels by classical microbiological methods [30].

### DNA preparation

Genomic bacterial DNA was extracted and purified from collected sera (*n* = 520) as well as bacterial isolates using DNeasy Blood and Tissue Kit (QIAGEN, Germany) according to the instructions of the manufacturer using a QIAcube pipettor. DNA concentration was determined photometrically using a Nano Drop ND-1000 UV-Vis spectrophotometer (Nano-Drop Technologies, Wilmington, DE, USA).

### PCR amplification

The extracted DNA was used for performing rt- PCR to detect the genus specific *Brucella* cell surface salt extractable *bcsp31* kDa protein gene, *B. abortus alk*B gene and *B. melitensis BME*I1162 gene [31]. The primers and TaqMan probes utilized for the assay were shown in Table 1. The rt- PCR assay was prepared using the TaqMan™ Environmental Master Mix (Applied Biosystems, New Jersey USA) containing the following components per reaction: 12.5 μl TaqMan™ Environmental Master Mix (Applied Biosystems), 0.5 μl of each primer (0.2 μM) and 0.25 μl of each probe (0.1 μM). Two  μl of bacterial DNA was used as target and nuclease-free water sum up to a total reaction volume of 25 μl. Negative Template Controls (NTC) that contained 2 μl of water instead of DNA and positive controls that contained DNA of *Brucella* were included in each run to detect any amplicon contamination or amplification failure. The rt - PCR reaction was performed in duplicate in optical 96-well microtitre plates (q PCR 96-well plates, Micro Amp TM, Applied Biosystem) using a Mx3000P thermocycler system (Stratagene, La Jolla, Canada) using the following reaction condition; initial denaturation at 95 °C for 10 min, followed by 50 cycles of 95 °C for 25 s and 57 °C for 1 min. The samples scored positive confirmed by visual inspection of the graphical plots showing cycle numbers versus fluorescence values. A sample with a fluorescence signal 30 times greater than the mean standard deviation in all wells over cycles 2 through 10 was considered a positive result, whereas a sample yielding a fluorescence signal less than this threshold value was considered a negative result. Cycle threshold values below 39 cycles were interpreted as positive.

**Table 1:** Oligonucleotide primers and probes used in the real-time PCR assay for the detection of *Brucella spp*., *B. abortus*, and *B. melitensis*

Target	Primer		
*Brucella* spp.	5`GCTCGGTTGCCAATATCAATGC 3`	Forward	Jena Bioscience GmbH, Germany
	5`GGGTAAAGCGTCGCCAGAAG 3`	Reverse	
	AAATCTTCCACCTTGCCCTTGCCATCA 6-FAM/BHQ1	Probe	
*B. abortus*	5`GCGGCTTTTCTATCACGGTATTC 3`	Forward	
	5`CATGCGCTATGATCTGGTTACG 3`	Reverse	
	CGCTCATGCTCGCCAGACTTCAATG HEX/BHQ1	Probe	
*B. melitensis*	5`AACAAGCGGCACCCCTAAAA 3`	Forward	
	5`CATGCGCTATGATCTGGTTACG 3`	Reverse	
	CAGGAGTGTTTCGGCTCAGAATAATCCACA CY5/BHQ2	Probe	

*6-FAM* 6-carboxyfluorescein, *HEX* 6-hexachlorofluorescein, *BHQ1* Black Hole Quencher 1, *BHQ2* Black Hole Quencher 2

### Molecular identification of bacterial isolates

Species identification of *Brucella* isolates was performed by using AMOS PCR [32]. The “Bruce-ladder PCR” was applied to identify the vaccine strain [33]. MLVA was also done to genotype the obtained isolates according to the previously described method using the MLVA-16 genotyping protocol [34]. Repeat numbers were calculated based on allele sizes measured by capillary electrophoresis (Applied Biosystems® 3130 Genetic Analyzer) according to the previously described procedure [35].

## Results

*Brucella* infection was diagnosed in the farm by using various serological and molecular techniques. The detailed results of serology as well as molecular diagnostic assays were reported in Tables 2 & 3. Briefly, one hundred twenty three samples (47.9 %) were found to be negative by using all serologic tests and rt-PCR; while 6.6 % (*n* = 17) yield positive by all serological tests in combination with rt-PCR at genus and species levels. ELISA, RBT and FPA also revealed the spread of *Brucella* field strains within the herd in 2013; their respective percentages were 11.3, 9.3 and 8.6 (Table 2). However, 36.96 % of the tested samples (*n* = 95) were seronegative but gave positive by rt-PCR where the only species identified was *B. abortus*.

**Table 2:** Detailed results of different serological tests and real time PCR for the detection of *Brucella* infection among cattle population during two years study period

	*n*	RBT	ELISA	FPA	Bcsp31 PCR (*Brucella* genus)	AlkB PCR (B. abortus)
Animals examined in 2013 (*n* = 257)	123	neg	neg	neg	neg	
	95	neg	neg	neg	pos	pos
	17	pos	pos	pos	pos	pos
	6	neg	pos	neg	pos	pos
	2	neg	pos	pos	pos	pos
	4	neg	neg	sus	neg	na
	5	pos	neg	neg	neg	na
	2	pos	pos	pos	neg	na
	2	neg	pos	sus	neg	na
	1	neg	neg	pos	neg	na
Animals examined in 2014 (*n* = 263)	1	neg	neg	sus	neg	na
	2	neg	pos	pos	neg	na
	1	neg	pos	sus	neg	na
	259	neg	neg	neg	neg	

*n* number, *neg* negative, *pos* positive, *susp* suspicious, *na* not applicable

**Table 3:** Characterisation of *Brucella* isolates of tissue specimens collected from an aborted fetus

origin	Conventional methods	*Brucella*-PCR	Amos-PCR	Ladder-PCR
Liver	*B. abortus*	Positive	*B. abortus*	*B. abortus* vaccine strain
Spleen	*B. abortus*	Positive	*B. abortus*	*B. abortus* field strain
Abomasal contents	*B. abortus*	Positive	*B. abortus*	*B. abortus* field strain
Genital organ	*B. abortus*	Positive	*B. abortus*	*B. abortus* field strain

Four *Brucella* isolates were recovered from the collected four specimens of the aborted fetus. These isolates were typed as one vaccine strain (from liver specimens) and three isolates of *B. abortus* field strain (from spleen, abomasum and genital organs) by using Bruce-ladder PCR (Table 3) while *B. abortus* was confirmed by AMOS PCR. The three field strain isolates showed the same MLVA-16 genotype. *B. abortus* was also identified in the sample of the farm veterinarian on the basis of a combination of serological tests (RBT, ELISA and FPA) and rt-PCR assay. Cows that were positive in at least two serological tests or one serological test plus the rt-PCR (*n* = 27) were removed from the farm in July 2013 while RB51 vaccine was further administered to all non pregnant heifers and parturient cows (30-45 days post calving). In May 2014, two hundred and sixty three samples gave negative results by using rt-PCR, RBT, ELISA and FPA; while three samples were seropositive.

## Discussion

Among the bacterial zoonoses affecting dairy cattle in developing countries, bovine brucellosis is considered the most prevalent infection when the applied control programs have failed. The infection poses not only a risk to animals, but also represents a zoonosis with debilitating illness and severe complications in humans. In Egyptian control programs for brucellosis, RB51 *B. abortus* vaccine is believed to be cleared from the blood stream within three days but can sporadically induce late term abortions if administered to pregnant cows [36]. The agent can then persist in infected tissues of the cow and fetus/calf. The presence of *Brucella* DNA in blood samples of these rare cases was supposed to be due to intermittent bacteremia whereas the organism is located intracellularly and may result in a persistent infection for long time [37]. In the present study, an unexpected high number of rt-PCR positive blood samples were detected five months after routine vaccination. The ninety five seronegative cows with positive rt-PCR results may have had circulating rough RB51 vaccine strain DNA in their blood as some researchers were able to detect RB51 vaccine strain DNA for twenty five days in the milk of vaccinated buffaloes although cultivation was possible only for 4 days [38]. Longer circulation of vaccine DNA in the blood stream is therefore a realistic possibility for the origin of DNA found. The twenty five serologically positive cows that showed *B. abortus* DNA in their blood stream may have been infected by the field strain. However, the seropositive animals with negative rt-PCR results (*n* =10) might represent animals at different stages of infection whenever the DNA is already cleared from the blood stream. The same suggestion was previously reported by Gwida et al. [39]

In general, the use of rt-PCR for the diagnosis of brucellosis from blood samples of ruminants has been discussed controversially. It has been supposed that the sensitivity of PCR is dependent on the status of disease i.e. the chronic infected animals are regularly misdiagnosed as no *B. abortus* DNA is present in respective samples [12]. The presence of *B. abortus* DNA was, however, demonstrated in blood samples from various infected animal species and humans but with varying sensitivities [9, 40, 41]. Recently, *B. abortus* and *B. melitensis* DNA were detected in bovine milk which was collected from apparently healthy animals by using species-specific IS711 rt-PCR [42]. It has been shown that rt-PCR could be an additional tool in an outbreak situation when serological methods are still negative or it might help to identify animals which are not detected by conventional serological methods [40].

The coincidence of vaccination and the apparent infection by a field strain in this herd which was serologically negative two months prior to the administration of the vaccine highlights the fact that RB51 vaccination cannot protect a herd (or single animals) from being infected with *B. abortus* in an endemic area. Vaccination may only reduce economical loss for the farmer. The sense of security conveyed by vaccination may even result in loss of awareness and impaired biosecurity. It was previously speculated that the vaccine strain can transmit from vaccinated animals to pregnant animals and cause abortion in very few cases [36]. Our findings support such suspicions where vaccine and field strain are isolated from the same fetus, suggesting that transmission of RB51 vaccine strain from non pregnant animals to unvaccinated pregnant animals has taken place. Our screening system made sure that most of the female animals used for reproduction on the farm were investigated with all techniques during the investigation period of two years. Vaccination and serological investigation with subsequent removal of reactors may result in the absence of abortions from July 2013 to May 2015. Although, in the routine RBT and BAPAT screening at May 2014, three cows tested positive which were later on reactive in ELISA and FPA but negative in rt-PCR. Simply, these animals could be undetected due to the stage of infection or could be re-contaminated via new introduction of a field strain. As a special problem for eradication of brucellosis, are those female calves that were borne to *Brucella* positive mothers. These calves may harbor the agent during their childhood and first pregnancy and seroconvert after they have calved [43–45]. In the setting of Egypt, such animals can run undetectable for approximately 20 months. We strongly advice to remove all seropositive animals from the herd although the fact that few false positive reactors may be culled.

The farm veterinarian contracted the infection with *B. abortus* during his daily work on the farm, when he was not aware that the disease was introduced only recently. This finding highlights the public health hazard of *Brucella* infection and could be an alarm for the potential risks for personnel elsewhere. We were unable to detect the source of infection, or the way through which the agent was introduced into the farm. It is an accepted fact that even very well managed farms in endemic areas can easily become contaminated [40]. It is obvious that prevention of damage in endemic areas should involve the practices of good farm management for biosecurity and biosafety. Therefore, we highlighted some findings made during this outbreak. In general, vaccination using a vaccine which does not interfere with serology is advised. However, current vaccines can cause abortions even if all recommendations are followed. The animal owner has to be made aware of this drawback of vaccines, although it has to be stressed that the benefit of vaccination outweighs its risks. Routine screening with a sensitive and specific test (validation of test has to be done in the region where it will be used) in short intervals has to be applied. It is worthy to note that no further abortions were recorded at the farm, since applying the strategy of test and slaughter. By the end of April 2014, 120 cows were culled from the farm, being either seropositive or having concomitant ailments including mastitis, laminitis and metritis. Special concern should be directed to females of a *Brucella* infected mother or those females which have unknown background, as such animals can be a carrier of *Brucella* and may seroconvert after abortion or calving only.

## Conclusion

We believe that RBT and BAPAT is a very useful combination of tests in Egypt, considering effectiveness and cost. If introduced, serological screening should be confirmed by ELISA and all reactors should be removed from the herd. All animals on the farm should be tested. We also suggest that PCR can be a good supplementary test for outbreak situations, as PCR can differentiate vaccine and field strains with high analytical sensitivity. We also recommend using RBT and ELISA in parallel in an outbreak situation to identify as many infected animals as possible during the initial screenings, and to repeat this test procedure for at least three successive negative tests with one month interval.
